# Editorial: Contemporary Families: Therapeutic Support for New Challenges

**DOI:** 10.3389/fsoc.2022.958271

**Published:** 2022-06-22

**Authors:** Sara Skandrani, Marion Feldman, Ricarda Nater-Mewes

**Affiliations:** ^1^UR 4430 Clinique Psychanalyse Développement, Université Paris Nanterre, Nanterre, France; ^2^Faculty of Psychology, University of Vienna, Vienna, Austria

**Keywords:** family relations, social adaptation, psychological outcomes, contemporary family, adoption, same-sex parenting, innovative mental health care

In the contemporary context, families are built and evolve in multiple, changing and more and more complex configurations. Women and men become parents in homosexual relationships, through international adoption, or across boundaries, in transnational spaces.

Children and adolescents grow up confronted with filiation, identity, or gender questions. Their family history can be multiple, rooted in different filiations and countries. When displacement and/or migration is part of their history, they sometimes have to cope with a difficult and painful past, on an individual but sometimes also collective level.

At the same time, new information and communication technologies and especially social media enter the families' homes, blurring boundaries between the outside world and the domestic sphere as well as possibly disrupting subjective development trajectories.

This Research Topic addresses the specificities of these evolutions in family life and their meaning for and impact on the psychological development of family members and their relations.

Same sex parent families were the focus of a review of qualitative and quantitative studies conducted by Siegel et al. They explored the psychological consequences of living in an ambiguous or hostile legal climate for children and parents, and how this may influence their family functioning.

Rabain explored relations in families with transgender adolescents, through the presentation of an innovative therapeutic approach, gathering parents, adolescents, as well as whole families together in different settings.

Another specific family configuration was studied by Skandrani, Moro et al. through their analysis of adoptive family relations when facing new forms of contacts with birth families, initiated by the latter through social media.

New information and communication technologies were also studied by Duriez, since they enter and revolutionize family relationships and organization. The author developed an Emotion Regulation Focused Family Therapy to face these new challenges.

Other studies published in this Research Topic questioned the contemporary social framework as well as the historical context in which these families evolve and change. They highlighted the impact of these contexts on the individual development as well as on family relations and their outcomes.

Feldman and Mansouri studied the consequences of breakdowns in filiation for children traumatically separated from their families in the Reunion Island and sent to foster families, adoptive families, and/or children's homes to mainland France in a specific public political context.

The specificities of the historical and transnational contexts were also studied by Grupp et al. with asylum seekers living in Germany and undocumented migrants and failed asylum seekers living in France. Family and transgenerational ties linked to spirituality appeared to be central in their understanding of their post-traumatic stress symptoms.

The new challenges families are confronted with have led mental health professionals to set up innovative therapeutic support and clinical settings, including group therapy, adoption therapy, multi-family therapy and transcultural therapy. The exploration of these new mental health care settings can promote the psychological, social and family outcomes in case of psychological suffering or difficulties in family relationships.

Two qualitative studies focused on an innovative mental health care setting, i.e., multi-family therapy, to address specific mental disorders families are confronted with. Baumas et al. explored the changes observed by adolescents with anorexia nervosa and their parents after a multi-family therapy, in terms of symptoms and family interactions, in focus groups. Roué et al. used semi-structured interviews to assess the benefits of multi-family therapy for adolescents with school refusal and their parents.

In order to promote mental health and prevent mental disorders or disruptions of family relations, an increasing number of clinical settings are provided for patient's family members. The contributions to this topic propose support groups for siblings of anorexia nervosa patients (Persico et al.), social support for donor families (Luo et al.), as well as couple therapy for partners of patients with chronic kidney disease (Riazuelo). These different mental health care settings promote the psychological, social, and family wellbeing in case of psychological suffering or difficulties in family relationships, and ultimately activate the families' resources.

## Author Contributions

All authors listed have made a substantial, direct, and intellectual contribution to the work and approved it for publication.

## Conflict of Interest

The authors declare that the research was conducted in the absence of any commercial or financial relationships that could be construed as a potential conflict of interest.

## Publisher's Note

All claims expressed in this article are solely those of the authors and do not necessarily represent those of their affiliated organizations, or those of the publisher, the editors and the reviewers. Any product that may be evaluated in this article, or claim that may be made by its manufacturer, is not guaranteed or endorsed by the publisher.

